# Influence of Group Modification at the Edges of Carbon Quantum Dots on Fluorescent Emission

**DOI:** 10.1186/s11671-019-3079-7

**Published:** 2019-09-02

**Authors:** Ju Tang, Jin Zhang, Yunfei Zhang, Yiming Xiao, Yanli Shi, Yunhua Chen, Lan Ding, Wen Xu

**Affiliations:** 1 School of Physics and Astronomy and Key Laboratory of Quantum Information of Yunnan Province, Yunnan University, Kunming, 650091 People’s Republic of China; 2 Key Laboratory of Materials Physics, Institute of Solid State Physics, Chinese Academy of Sciences, Hefei, 230031 People’s Republic of China

**Keywords:** Carbon quantum dot, Functional group modulation, Fluorescent emission

## Abstract

We present a detailed investigation on the effect of functional group modulation at the edges of carbon quantum dots (CQDs) on the fluorescence from the CQDs. The CQDs attached by N, S, and P elements are synthesized via pyrolysis of a mixture of citric acid and NH_3_H_2_O, H_2_SO_4_, and H_3_PO_4_, respectively. Thus, part of –COOH at the edges of CQDs can be converted into –C=O and functional groups such as –NH_2_, –SO_2_, –HSO_3_, and –H_2_PO_4_ can connect to the carbon bonds. We find that the formation of the N/S/P-CQDs can reduce the amount of –COOH that attaches to the edges of sp^2^-conjugated *π*-domains located at centers of these CQDs. This effect can result in the reduction of the non-radiative recombination for electronic transition in these CQDs. As a result, the quantum yield (QY) for fluorescence from the CQDs can be efficiently enhanced. We demonstrate experimentally that the QYs for N/S/P-CQDs can reach up to 18.7%, 29.7%, and 10.3%, respectively, in comparison to 9% for these without functional group modulation. This work can provide a practical experimental approach in improving the optical properties of fluorescent CQDs.

## Background

Carbon quantum dots (CQDs) are emerging nanomaterials [1] with superior fluorescent properties [2] and unique chemical, electronic, and optical properties [3]. In contrast to traditional dye molecules and semiconductor-based quantum dots, the CQDs are not only with the good light resistance and scintillation light bleaching resistance [4] but also with important features such as low toxicity, biocompatibility, low cost, high photostability [5], etc. Hence, the CQDs have been proposed as advanced electronic and optoelectronic materials for application in the areas such as optoelectronic devices [6], energy conversion [7], photocatalysis [8], sensors [9], bio-imaging [10], cell markers [11, 12], and drug delivery [13], to mention but a few. In recent years, the investigation of CQDs has become a hot and fast-growing field of research in scientific and industry communities.

At present, the most popularly employed experimental method for chemical synthesis of the CQDs is via bottom-up approach which can be applied to produce fluorescent CQDs simply, cheaply, and in large-scale quantity. In this method, small molecules of organic compounds or polymers are taken as carbon sources and are dehydrated and carbonized to realize the CQDs. During the preparation of the CQDs, the surface and edge of the CQDs are often attached by some chemical groups such as OH, COOH, C=O, and so on. The presence of these chemical groups can affect greatly the electronic and optical properties of the CQDs. In particular, the fluorescent characteristics of the CQDs depend sensitively on the presence of these groups [14]. From a viewpoint of physics, the chemical groups attached to the surface and edge of the CQDs can induce new kinds of surface and edge states into the CQD systems and, thus, can modify the electronic structure and the corresponding electronic and optical transition channels in the CQDs. In this case, the photoluminescence (PL) from the CQDs can be achieved via electronic transition between edge states and carbon states such as conjugate *π* states (or sp^2^ area). Thus, the mechanism of the PL emission from CQDs is very much akin to that for photo-induced light emission from impurity states in a doped semiconductor [15] in the following way: (i) the photons can be absorbed via electronic transitions from lower and occupied carbon electronic states to higher and empty states under the action of optical pumping, (ii) the photo-excited electrons can be relaxed into the electronic states in the edge states via non-radiative electronic transition events, and (iii) the PL emission can be achieved via electronic transitions from edge states to the lower carbon electronic states accompanied by the emission of photons. Therefore, the edge electronic states play a role like radiative impurity states in a semiconductor and the electrons can be combined with the holes for the luminescence. Generally, the sp^2^-conjugated *π*-domains are considered to be as the inherent centers for PL emission [16] and the aromatic sp^2^ carbon area of a CQD is surrounded by sp^3^ carbon (C–OH) states. Hence, the recombination of electron-hole pairs in the sp^2^ area and the electronic transitions among carbon and edge states can promote the PL emission from CQDs [17, 18].

The fluorescent quantum yield (QY) is a key parameter to measure the efficiency of photo-induced light emission from a material or a device, which is defined by the number of photons emitted relative to the number of photons absorbed. In the early years when the CQDs were discovered, the QY for chemically prepared CQDs was quite low (even less than 2%) [1]. How to improve the fluorescent QY for CQDs has been a central problem for fundamental research and for material application. In general, the QY for CQDs realized chemically via a bottom-up approach depends on the choice of the carbon source, synthesis technique, and edge modulation. More specifically, the intensity and frequency of the PL emission from CQDs are the consequences of the sample parameters of the CQDs, the presence of the functional groups or edge states, the interaction between electronic states in sp^2^-conjugated *π*-domains and in chemical groups, and the properties of the fluorophore in the CQDs [19]. In recent years, the fluorescent QY for CQDs has been largely improved [20]. Particularly, Lingam et al. examined the effect of the edge states induced in the synthesis of CQDs on the PL emission. They found that if the edges of the CQDs are damaged, the PL emission drops sharply to be even immeasurable [21]. A similar work by Kumar et al. has also demonstrated that the presence of the edge states is the key factor for PL from CQDs, and the origin of tunable heterogeneous PL is amino-functionalized for CQDs [22]. Tang et al. reported a simple microwave-assisted hydrothermal synthesis of the CQDs using glucose as the sole carbon source [23]. By simply extending the reaction time from 1 min to 9 min, they could adjust the size of the CQDs from 1.65 nm to 21 nm. Interestingly, they found that the PL from the CQDs was independent upon the size of the CQDs, where the CQDs with the sizes of 9.6 nm and 20 nm show roughly the same light absorption and emission behaviors. The results obtained from further research work by Lin et al. also indicate that the PL emission from CQDs depends weakly on the effect of quantum confinement of the sp^2^-conjugated *π*-domains [24] and the presence of the surface functional groups attached to the CQDs is the key factor for PL emission. Dong et al. realized the blue fluorescent CQDs with the disc-like nanosheets (the size of 15 nm and the thickness of 0.5–2.0 nm) by adjusting the degree of the carbonation of citric acid. They found that the PL of CQDs is independent on the excitation wavelength and the fluorescent QY can be up to 9.0% [25]. It should be noted that according to the relationship between the size of a CQD and the wavelength of the PL emission for a bare CQD [26], the CQDs with the size of about 2.25 nm can emit the blue fluorescence, while the CQDs with the size of 15 nm can only emit longer wavelength fluorescence. The strong blue PL emission from CQDs with the size of 15 nm [25] suggests once more that the presence of the edge states induced by chemical groups attached to the CQDs is mainly responsible for PL emission from CQDs. Therefore, the edge functionality of the CQDs can affect not only the fluorescence of CQDs, but also the physical and chemical properties of the CQDs in general [19].

It should be noticed that at present, the frequency of the fluorescent emission from CQDs cannot be easily controlled and modified artificially. Furthermore, the corresponding fluorescent QY has not yet reached the requirements for the application as practical devices. The results obtained from experimental [27–29] and theoretical [30, 33] research have confirmed that the edge passivation can effectively improve the electronic optical properties of CQDs. Passivation agents are widely used to adjust the fluorescent properties of CQDs [21–23]. Jing Liu et al. developed a one-step preparation of nitrogen-doped and surface-passivated carbon quantum dots [27]. They found that the QY of CQDs without surface passivation is usually quite low (QY < 10%), passivated CQDs show a QY of 37.4%. A similar work by Shen et al. researched the PEG-passivated CQDs have a QY of 28% [28], and Kwon et al. reported that hexadecyl amine (HDA)-passivated one yields 19–35% [29]. Dimos and co-workers also found that the edge passivation can effectively induce the electrons in the conduction band and increase the surface energy of the CQDs to prevent fluorescence decay or quenching caused by the aggregation of CQDs [30]. Furthermore, the fluorescent generation from CQDs fabricated via chemical reaction or bottom-up approach is mainly induced by the presence of the radiative functional groups or fluorophore attached to the edge of the CQDs [31]. Chemically, these functional groups can be modified via putting the CQDs into different chemical solutions. The fluorescence of these chemically modified CQDs can be achieved via the excitonic emission accompanied by electronic recombination and separation of electron-hole pairs trapped on the edge of CQDs [32]. Thus, the frequency of the PL emission can be tuned via selecting different edge groups to form required edge states.

The prime motivation of this study is to explore how the fluorescent properties of the CQDs can be modified in different chemical solutions for their passivation or edge functionality. Recently, we had produced CQDs from tofu wastewater without adding any toxic substances and revealed the luminescence mechanism [33]. We found that different colors of the fluorescent emission from these CQDs can be achieved via putting these dot materials in water or in NaOH solution [33]. We had also fabricated the CQDs from fresh lemon juice and applied them for cellular imaging [34]. It was found that the presence of oxygen-containing groups on the surface and edge of the CQDs is mainly responsible for the fluorescence of the CQDs [34]. Our attention of the present study is mainly focused on how to achieve an effective way in improving the quantum yield of fluorescence from CQDs.

## Method

### Synthesis of N-, S-, and P-CQDs

In this work, citric acid (C_6_H_8_O_7_, 99.5%), sodium hydroxide (NaOH, 96%), ammonia solution (NH_3_ H_2_O, 25–28%), sulfuric acid solution (H_2_SO_4_, 98%), phosphoric acid solution (H_3_PO_4_, 85%), and deionized water were used to fabricate the CQDs and to modify the edge states.

**Fig. 1:**
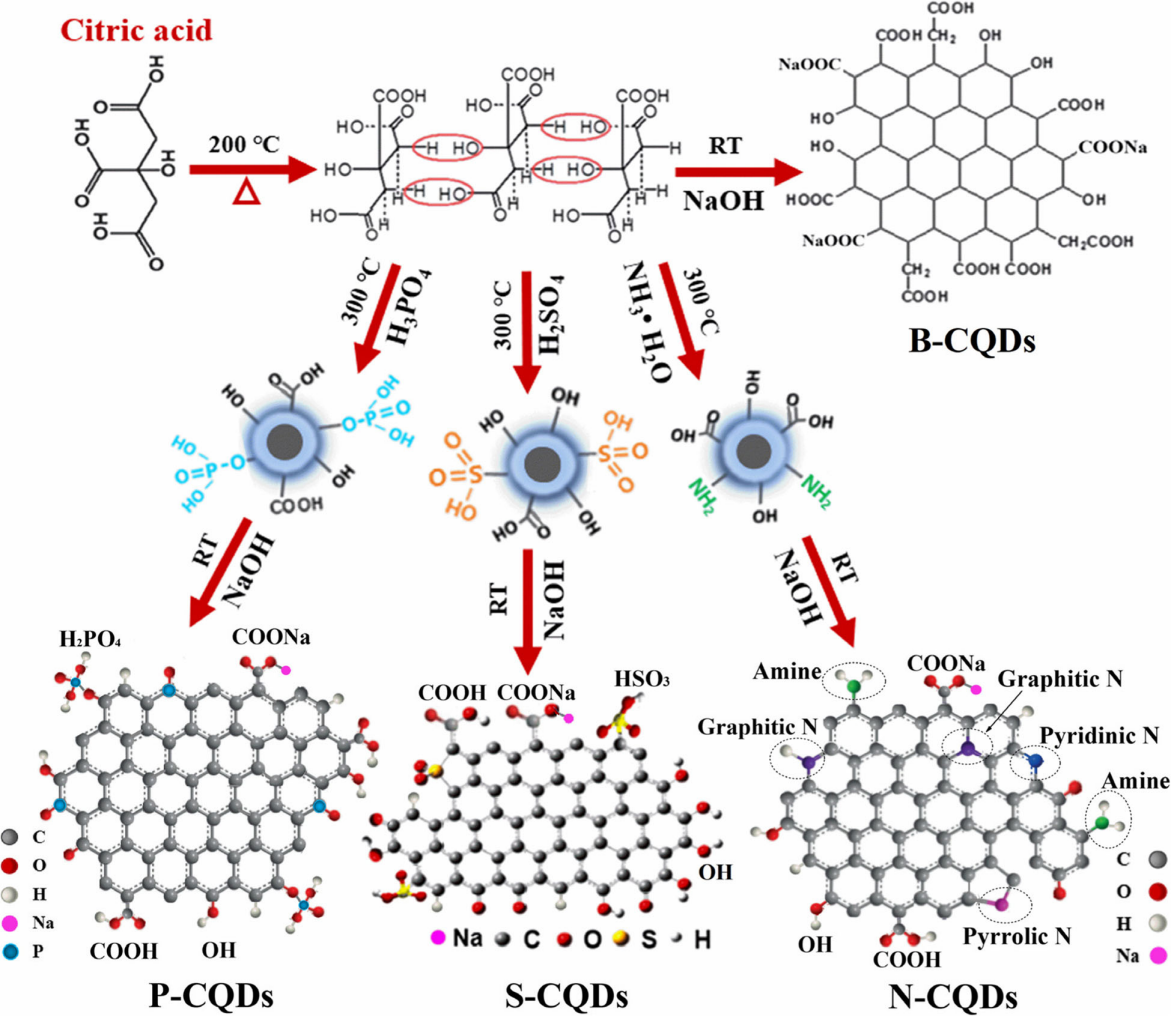
Schematic synthesis processes of CQDs and corresponding N-, S-, and P-CQD structures

The B-CQDs were synthesized by pyrolysis of citric acid as sole carbon source. The N/S/P-CQDs were fabricated by pyrolysis of ammonia, sulfuric acid, and phosphoric acid together with citric acid, respectively. The principle and the experimental processes for the realization of the CQDs and corresponding N/S/P-CQDs are shown in Fig. 1. Through the pyrolysis of the citric acid, the B-CQDs can be fabricated via inter-molecular dehydration, carbonization, and condensation reaction. This approach can form (i) the aromatic structure (namely the sp^2^ carbon states with C–C and C=C bonds) with conjugate area as the core of the CQDs, (ii) the edges of the CQDs attached by hydroxyl (OH) and carboxyl (COOH/–O–C=O), and (iii) the sp^3^ carbon (C–C–OH/–C–O) area which can be passivated in, e.g., NaOH solution. The N-CQDs can be achieved via pyrolysis process of the mixed solution of citric acid and NH_3_H_2_O. In such a case, the dehydrogenation reaction among adjacent groups with carboxyl can promote the formation of pyrrolic N in graphene skeleton of the CQDs. The edges of the N-CQDs are then attached with extra chemical groups such as –C–N and –NH_2_ [35]. Similar to the realization of the N-CQDs, S-CQDs, and P-CQDs can be fabricated via the pyrolysis of the mixed solution of citric acid and H_2_SO_4_ and H_3_PO_4_, respectively. The inter-molecular dehydration, carbonization, and condensation reaction can form graphene skeleton of the CQDs with the connection to chemical groups such as –SO_2_, –HSO_3_, and –H_2_PO_4_. Furthermore, S and P atoms are likely to attach to the edge of sp^2^ carbon conjugate area to form the edge defects. The material structures of N/S/P-CQDs are shown in Fig. 1. In the present study, N/S/P-CQDs are dispersed in NaOH solution at room temperature to make them passivation. Thus, we can convert part of the –COOH at the edge of the CQDs into –COONa and reduce the amount of –COOH at the edge of sp^2^-conjugated *π*-domains. Consequently, the group modification at the edge of the CQDs can be achieved.

More specifically, the fluorescent N/S/P-CQDs are prepared in the following ways: (i) 2 g analytically pure citric acid was added into 60 ml NH_3_H_2_O (pH = 7.5), 60 ml H_2_SO_4_ (pH = 3), and 60 ml H_3_PO_4_ (pH = 3), respectively. (ii) We place the mixed solution onto the heating platform for constant heating at 300 °C, where the heating time is for 20–30 min. (iii) 50 ml sodium hydroxide solution (NaOH) is added into the beaker after the beaker cooling down naturally till room temperature. (iv) The mixture is magnetically stirred for 10 min and ultrasonically shocked for 10 min and further centrifuged at a speed of 12,000 r/min for 10–30 min. Then, the supernatant is taken, in which N/S/P-CQDs modified by ammonia, sulfuric acid, and phosphoric acid solutions, respectively, and passivated by groups such as –OH, –COOH, –C=O, –COONa, –NH_2_, –SO_2_, –HSO_3_, and –H_2_PO_4_ can be obtained in NaOH solutions. In the preparation of N/S/P-CQDs, except that the N/S/P-CQDs were modified by NH_3_H_2_O, H_2_SO_4_, and H_3_PO_4_, respectively, the other experimental conditions were roughly the same. We used the same amount of citric acid to dissolve into NH_3_H_2_O, H_2_SO_4_, and H_3_PO_4_, respectively, for pyrolysis and then added the same amount and equal concentration of sodium hydroxide solution when the substances in the beaker were almost dry. This can ensure that the density of the CQDs in NaOH solution is almost the same.

## Measurements

In this work, the morphology and microstructure of the CQDs were observed by using the transmission electron microscopy (TEM, JEM 2100) at an accelerating voltage of 300 kV. The X-ray photoelectron spectroscopy (XPS) was applied to characterize the samples, by using PHI5000 Versa Probe II photoelectron spectrometer with Al Kα at 1486.6 eV. The ultraviolet-visible (UV-Vis) absorption spectrum of the CQDs was measured by a UV-Vis spectrophotometer (Specord200, Germany). The PL emission from the CQDs was measured using a standard PL system (IHR320, Jobin Yvon, USA) at room temperature. The fluorescent QY of the CQDs is evaluated on the base of the PL data.

## Results and Discussions

### The Characterization of Samples

**Fig. 2:**
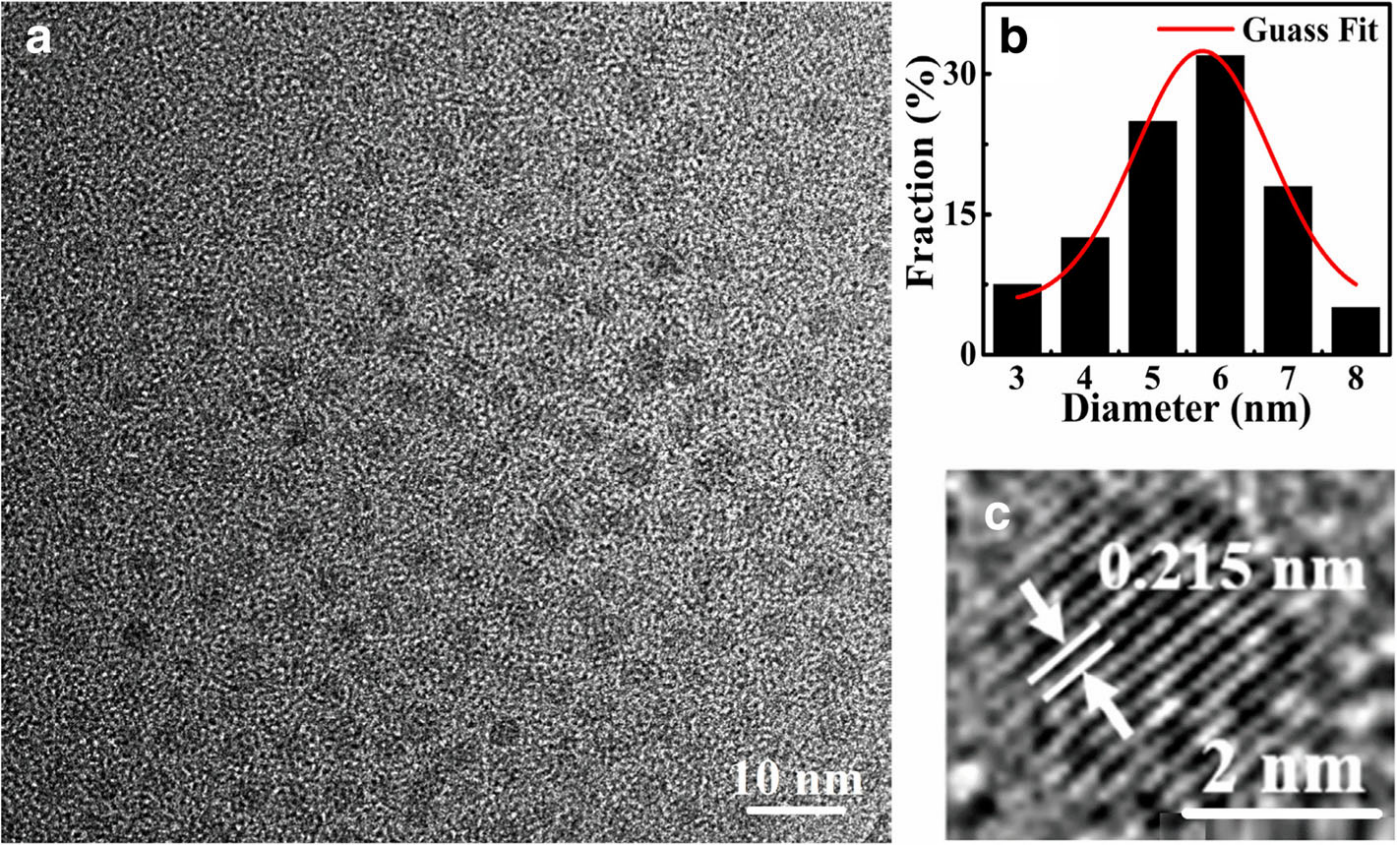
TEM images of S-CQDs in NaOH (pH = 12) solution in **a** and their lattice fringes in **c**. The diameter distribution of the S-CQDs is shown in **b**

The morphology and structures of as-synthesized CQDs were investigated by TEM. Figure 2 shows the TEM images (a) and the diameter distributions (b) of S-CQDs. It can be clearly seen that the S-CQDs are circular-like sheets and are dispersed uniformly in NaOH solution. Through a statistical average of the TEM images for the CQDs, we find that the size distribution of S-CQDs is mainly located in 3–8-nm range and the average size of the CQDs is about 5.73 nm. These results are obtained by statistical analysis of more than 300 CQD particles using the Image *J* software. The results shown in Fig. 2 c indicate that these S-CQDs are highly crystallized with typical lattice structure of carbon. The lattice fringes of the CQDs are clear and the corresponding lattice spacing is about 0.215 nm consistent with the (100) facet of graphene [32].

**Fig. 3:**
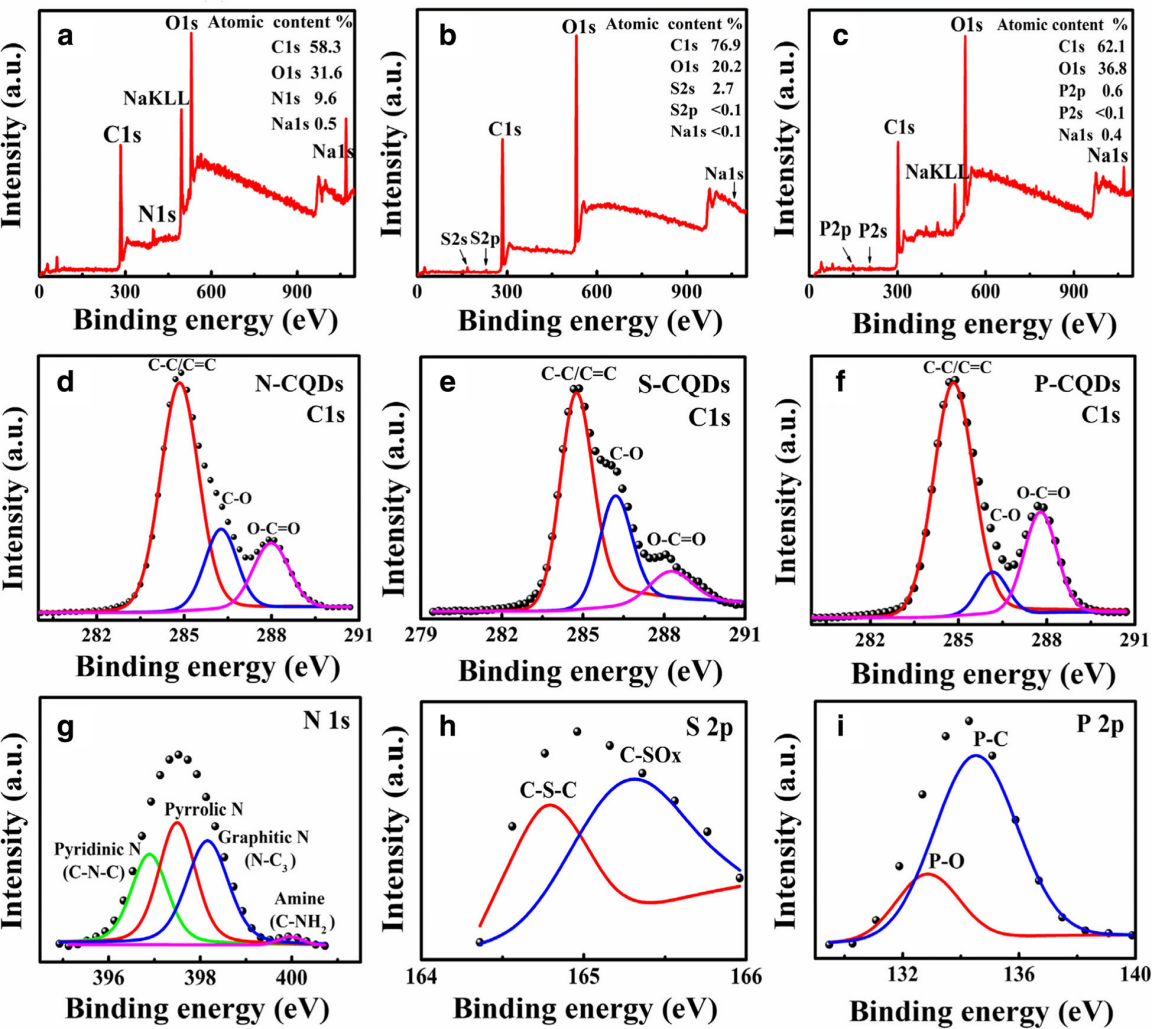
The full-scan XPS spectra in **a**/**b**/**c** and the high-resolution spectra of C1s in **d**/**e**/**f** and N1 s/S2p/P2p in **g**/**h**/**i** for, respectively, the N/S/P-CQDs

In the present study, we apply the X-ray photoelectron spectroscopy (XPS) for the measurement and examination of the edge functional groups attached to the CQDs. We use glass sheets as the substrates and coat the samples on the glass sheets for the measurements. In Fig. 3, we show the XPS spectra for N-, S- and P-CQDs. As shown in Fig. 3 a, the full-scan XPS spectrum for N-CQDs indicates that the atomic percentage of N1s is 9.6%. Figure 3 b and c indicate that (i) the S- and P-CQDs show response peaks for S2s (at 169 eV) and P2s (at 133 eV), respectively, in contrast to CQDs prepared without functional group modification and (ii) the atomic percentages of S2s for S-CQDs and of P2p for P-CQDs are 2.7% and 0.6%, respectively. The main reason for a lower content of P in P-CQDs than S content in S-CQDs is that P has a relatively larger atomic radius (proton number of nuclei is 15) than S has (proton number of nuclei is 16). Thus, P atoms are relatively less likely to be absorbed by the chemical bonds on the surface of CQDs than S atoms are. In comparison with Fig. 3 a and c for the XPS spectra for N- and P-CQDs, Fig. 3 b shows that the percentage of C1s atoms in S-CQDs is much higher (76.9%) and the proportions of O1s atoms (20.2%) and impurity atoms (S, Na) are very low. These results indicate that there are fewer defects in the sp^2^ carbon area in S-CQDs. Shown in Fig. 3 d are the high-resolution spectra of C1s exhibits three typical peaks, respectively, at 284.8 eV, 286.6 eV, and 288.5 eV for N-CQDs, which indicate that the sp^2^ carbon (C–C/C=C) area has a good lattice structure [14, 25, 36]. Furthermore, sp^3^ carbon (C–O/C–N, 286.4 eV) and carboxyl (O–C=O/COOH, 288.1 eV) have similar binding energy [33], suggesting that there are similar amounts of hydroxyl (C–O/C–OH) and carboxyl (O–C=O/COOH) around the sp^2^ carbon area. The corresponding high-resolution spectra of C1s for S- and P-CQDs are shown in Fig. 3 e and f. As shown in Fig. 3 e, the peak of sp^3^ carbon (C–O) is very strong and the peak of hydroxyl (O–C=O/COOH) is weak for S-CQDs, indicating that the amount of hydroxyl (C–O/C–OH) are much higher than carboxyl (O–C=O/COOH) around the sp^2^ carbon area for S-CQDs. In addition, Fig. 3 h indicate that the amount of hydroxyl (C–O/C–OH) is less than carboxyl (O–C=O/COOH) around the sp^2^ carbon area for P-CQDs. Comparing with Fig. 3 d, e, and f, we find that the peak of hydroxyls (C–O/C–OH) is the strongest one while the peak of hydroxyls (O–C=O/COOH) is the weakest one for S-CQDs. When the amount of hydroxyl groups is increased, the amount of carboxyl groups is reduced, and vice versa, when the amount of carboxyl groups is increased, the amount of hydroxyl groups is decreased.

In Fig. 3 g, the high-resolution spectra of N1s show four peaks related to pyridinic nitrogen (pyridinic N, 396.9 eV), pyrrolic nitrogen (pyrrolic N, 397.6 eV), graphite nitrogen (N–C_3_, 398.5 eV, located in the center of sp^2^ carbon) and the amide group (C–NH_2_, 399.9 eV, located at the edge of sp^2^ carbon), respectively [22, 37–39]. These results verify the material structure of N-CQDs shown in Fig. 1. In N-CQDs, the fluorophores are formed by the hybridization of edge functional groups such as C–OH and C–NH_2_ with sp^2^-conjugated *π*-domains [40], which can play a role in enhancing the fluorescence of N-CQDs. The high-resolution spectrum of S2s and P2p corresponding to XPS results for S- and P-CQDs are shown in Fig. 3 h and i, it implies that the edges of S-CQDs and P-CQDs are attached by some chemical bonds such as C–S–C (164.8 eV) [41], C–SO_*X*_ (*X* = 2, 3, 4, 165.1 eV) [42], P–C (135.0 eV) [43], and P–O (132.7 eV) [44]. Thus, the chemical bonds such as C–SO_2_, –HSO_3_, C–P–C, –H_2_PO_4_, etc. can be formed at the edges of the S- and P-CQDs, as shown in Fig. 1.

**Fig. 4:**
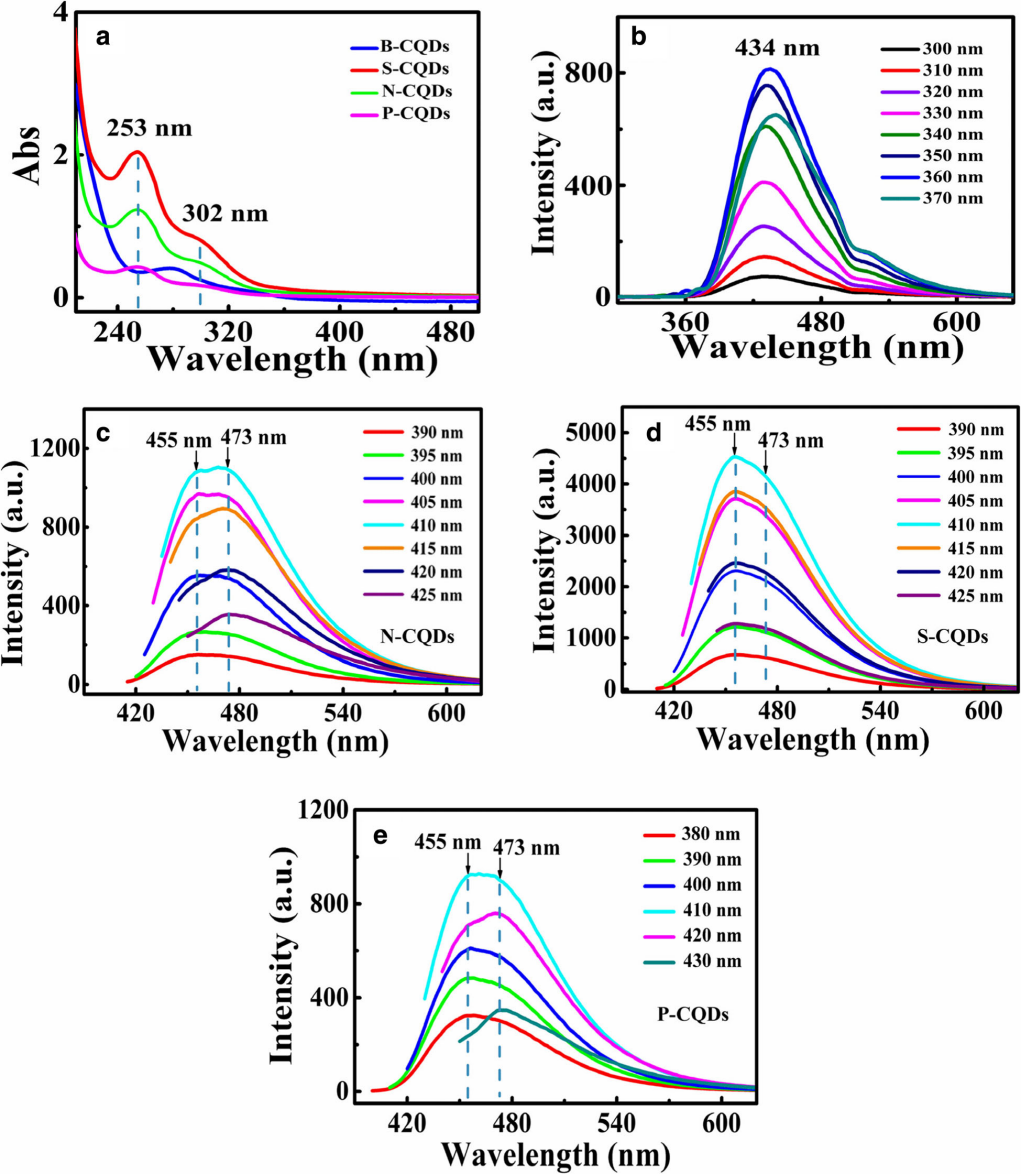
The spectra of UV-Vis absorption by B- and N/S/P-CQDs in **a**. The PL spectra of **b** B-CQDs, **c** N-CQDs, **d** S-CQDs, and **e** P-CQDs with different excitation wavelengths as indicated. These results are obtained at room temperature

Fig. 4 shows the absorption and emission spectra measured from the B- and N/S/P-CQDs in NaOH solutions. Shown in Fig. 4 a are the UV-Vis absorption spectra of B/N/S/P-CQDs. The absorption spectrum of B-CQDs exhibits UV absorption peaks at 278 nm, while UV-Vis absorption spectrum of N-, S-, and P-CQDs depicts two clear absorption bands. The absorption peak at 253 nm is attributed to *π*–π* transition of aromatic C=C bond and the shoulder at 302 nm corresponds to *n*–π* transition of C=O bond [40]. The C=C bonds come from sp^2^-conjugated domains in the cores of N/S/P-CQDs, while C=O bonds originate from the numerous electron-withdrawing oxygen-containing groups such as carboxyl and carbonyl groups existing at the edge sites of N/S/P-CQDs. The two absorption peaks reveal the existence of sp^2^-conjugated structures and oxygen-containing functional groups (C=O and O–C=O/COOH) in N-, S-, and P-CQDs.

Figure 4 b–e show the PL spectra of B- and N/S/P-CQDs. As we can see, the strong PL emission can be observed in these CQDs. We find that the intensity of the PL emission first increases then decreases with increasing excitation wavelength. This effect has been found in most nano-structured materials (see, e.g., Ref. [45]). As has been pointed out by Ref. [38], the dependence of the intensity of the PL emission upon excitation for CQDs is mainly originated due to heterogeneous surface states and the sizes and electronic properties of the heteroatoms. For the case of the CQDs with surface group modulation, the presence of the surface states can alter the band gap and radiative energy states of the CQDs. Under relatively shorter wavelength excitation, the electrons are pumped into higher energy states in conduction band further away from radiative electronic states induced by the surface modulation. Thus, the possibility for photon emission from CQDs is reduced by shorter wavelength excitation. Namely, the intensity of the PL emission increases with excitation wavelength in a shorter wavelength regime. In a relatively long wavelength regime, longer wavelength excitation implies that relatively less electrons can be pumped into the conduction band in CQDs. As a result, the intensity of the PL emission decreases with increasing excitation wavelength in a long excitation wavelength regime. Furthermore, the PL emissions of N/S/P-CQDs consist of two overlapping spectral bands [25]. The double PL peaks can be measured respectively at about 455 nm and 473 nm via 410-nm wavelength excitation. This is a consequence of the PL emissions from N/S/P-CQDs modified by different functional groups to affect the radiative recombination of electron-hole (e-h) pairs formed by sp^2^-conjugated *π*-domains and edge state groups [32, 40]. The possible mechanism proposed here is that (i) the photo-excitation of electrons in N/S/P-CQDs is achieved via *π*–π* transitions from sp^2^-conjugated *π*-domains to *n*–π* transitions, (ii) the electrons are relaxed from *n*–π* states to C=O energy levels via non-radiative transition channels, and (iii) the radiative recombination for holes in discrete sp^2^-related states and electrons in edge states can be induced by abundant functional groups [46].

In Fig. 4 b–e, under the same experimental conditions, we find that the fluorescence intensity of N/P/S-CQDs were significantly higher than that of B-CQDs. The intensity of PL emission from S-CQDs is the strongest, followed by N-CQDs, and the PL emission from P-CQDs is the weakest. This finding corresponds to the results shown in high-resolution spectra of C1s for N/S/P-CQDs (see Fig. 3d, e, f). The intensity of PL emission increases with increasing the peak height of C–O (C–OH) and with decreasing the peak height of O–C=O (COOH). Therefore, it is reasonable to believe that N/S/P-CQDs with various edge groups, such as C–OH, COOH, C=O, and C–H, can induce different kinds of edge states and influence the intensity of its photoluminescence. Most interestingly, we find that the peak positions for PL emission from N/S/P-CQDs depend on the excitation wavelength, which implies that the radiative electronic states induced by functional groups attached to the edges of the CQDs are quite stable.

As mentioned above, sp^2^ carbon area of S-CQDs has fewer defects so that the inherent light emission from S-CQDs is the strongest, compared with N- and P-CQDs. After using the formula for evaluating the fluorescent quantum yield [33], we obtain that the QYs for N/S/P-CQDs are 18.7%, 29.7%, and 10.3%, respectively. Thus, the largest QY can be achieved for S-CQDs, followed by N- and P-CQDs. As we know, the QY of CQDs is a consequence of competing process between radiative electronic transition and non-radiative traps [32]. The hydroxyl (C–O/C–OH) at the edges of the CQDs can be hybridized synergistically with electronic states in sp^2^-conjugated *π*-domains to form the fluorophores, while those carboxyl (–COOH) can play a role like non-radiative recombination center at the edge of the CQDs [40]. Therefore, the QY of CQDs is affected by the C–O/C–OH and O–C=O/COOH groups. Moreover, we find that using sulfuric acid for modification of the functional groups at the edge of S-CQDs can affect significantly the enhancement of PL emission from S-CQDs, compared with N- and P-CQDs. The QY of 29.7% for S-CQDs, realized in this study, is larger than 18.7% for N-CQDs, 10.3% for P-CQDs, and 9% for CQDs [23] prepared without ammonia, sulfuric acid, or phosphoric acid.

In this work, the N/S/P-CQDs are synthesized by pyrolysis of these mixtures of citric acid and NH_3_H_2_O, H_2_SO_4_, and H_3_PO_4_, subsequently dispersed in the NaOH solutions. It can promote inter-molecular dehydration, carbonization, and condensation reaction for citric acid. Carboxyl (–COOH) at the edges of the CQDs can be converted into carbonyl (–C=O) [19], and the edges of N/S/P-CQDs can be attached by chemical groups such as –OH, –COOH, –C=O, –NH_2_, –SO_2_, –HSO_3_, and –H_2_PO_4_. Therefore, the formation of the N/S/P-CQDs can reduce the amount of –COOH attached to the edges of sp^2^-conjugated *π*-domains and can result in the reduction of non-radiative recombination [47]. Consequently, the fluorescent QY of CQDs can be efficiently enhanced.

## Conclusions

In this study, we have developed an effective experimental method to enhance the fluorescent quantum yield of CQDs. N/S/P-CQDs have been synthesized by pyrolysis citric acid and using, respectively, ammonia, sulfuric acid, and phosphoric acid for edge group modifications. The results have shown that the presence of the edge functional groups can play a significant role in generating and enhancing the fluorescence from these CQDs. Especially, the hydroxyl (C–O/C–OH) groups at the edges of sp^2^-conjugated *π*-domains can affect significantly the fluorescent quantum yields of the CQDs. Nevertheless, the attachment of carboxyl (O–C=O/–COOH) groups to the edges of sp^2^-conjugated *π*-domains leads mainly to non-radiative recombination centers, which can weaken the PL emission from the CQDs. In the present study, the QYs for N/S/P-CQDs can reach up to 18.7%, 29.7%, and 10.3%, respectively. These values are much higher than that of 9% for CQDs prepared without functional group modification. The most important conclusion we draw from this study is that the group modification at the edges of the CQDs by sulfuric acid can affect strongly the fluorescence emission and QY of the CQDs.

## Data Availability

The datasets generated during and/or analyzed during the current study are available from the corresponding authors on reasonable request.

## Abbreviations

CQDs: Carbon quantum dots
PL: Photoluminescence
QY: Quantum yield
TEM: Transmission electron microscope
UV-Vis: Ultraviolet visible
XPS: X-ray photoelectron spectroscopy
XRD: X-ray diffraction

## References

[1] Xu X, Ray R, Gu Y (2004). Electrophoretic analysis and purification of fluorescent single-walled carbon nanotube fragments. Journal of the American Chemical Society.

[2] Sun YP, Zhou B, Lin Y (2006). Quantum-sized carbon dots for bright and colorful photoluminescence. Journal of the American Chemical Society.

[3] Novoselov KS, Geim AK, Morozov SV (2004). Electric field effect in atomically thin carbon films. Science..

[4] Ding Y, Zhang F, Xu J (2017). Synthesis of short-chain passivated carbon quantum dots as the light emitting layer towards electroluminescence. RSC Adv.

[5] Chen Z, Wang X, Li H, Li C, Lu Q, Yang G, Long J, Meng L (2015). Controllable and mass fabrication of highly luminescent N-doped carbon dots for bioimaging applications. RSC Adv.

[6] Bourlinos AB, Stassinopoulos A (2008). Surface functionalized carbon genic quantum dots. Small No..

[7] Wang Z, Yuan F, Li X, Li Y, Zhong H, Fan L, Yang S (2017). 53% efficient red emissive carbon quantum dots for high color rendering and stable warm white-light emitting diodes. Adv. Mater.

[8] Wang F, Liu C-y (2011). White light-emitting devices based on carbon dots electroluminescence. Chem Commun.

[9] Wang K, Gao ZC, Gao G (2013). Systematic safety evaluation on photo luminescent carbon dots. Nanoscale Res Lett..

[10] Bhunia SK, Saha A, Maity AR, Ray SC, Jana NR (2013). Carbon nanoparticle-based fluorescent bioimaging probes. Scientific Reports volume.

[11] Tao H, Yang K, Ma Z, Wan J, Zhang Y, Kang Z, Liu Z (2013). In vivo NIR fluorescence imaging, biodistribution, and toxicology of photoluminescent carbon dots produced from carbon nanotubes and graphite. Small.

[12] Wu ZL, Zhang P, Gao MX (2013). One-pot hydrothermal synthesis of highly luminescent nitrogen-doped amphoteric carbon dots for bioimaging from Bombyx moil silk-natural proteins. J Mater Chem B.

[13] Bhunia SK, Pradhan N, Jana NR (2014). Vitamin B1 derived blue and green fluorescent carbon nanoparticles for cell-imaging application. ACS Appl Mater Interfaces.

[14] Mondal TK, Gupta A, Shaw BK (2016). Highly luminescent N-doped carbon quantum dots from lemon juice with porphyrin-like structures surrounded by graphitic network for sensing applications. RSC Adv.

[15] Ajimsha RS, Anoop G, Aravind A (2008). Luminescence from surfactant-free ZnO quantum dots prepared by laser ablation in liquid. Electrochemical and Solid-State Letters.

[16] Chien C-T, Li S-S, Lai W-J (2012). Tunable photoluminescence from graphene oxide. Angew. Chem. Int. Ed.

[17] Zhu S, Tang S, Zhang J, Yang B (2012). Control the size and surface chemistry of graphene for the rising fluorescent materials. Chem. Commun..

[18] Wilson WL, Szajowski PF, Brus LE (1993). Quantum confinement in size-selected, surface-oxidized silicon nanocrystals. Science.

[19] Dan Q, Zheng M, Zhang L, Zhao H (2014). Formation mechanism and optimization of highly luminescent N-doped graphene quantum dots. Sci. Rep.

[20] Chen C-F, Park C-H, Boudouris BW (2011). Controlling inelastic light scattering quantum pathways in graphene. Nature.

[21] Lingam K, Podila R, Qian H (2013). Evidence for edge-state photoluminescence in graphene quantum dots. Adv. Funct. Mater.

[22] Sandeep Kumar G., Roy Rajarshi, Sen Dipayan, Ghorai Uttam Kumar, Thapa Ranjit, Mazumder Nilesh, Saha Subhajit, Chattopadhyay Kalyan K. (2014). Amino-functionalized graphene quantum dots: origin of tunable heterogeneous photoluminescence. Nanoscale.

[23] Tang L, Ji R, Cao X, Lin J, Jiang H, Li X (2012). Deep ultraviolet photoluminescence of water-soluble self-passivated graphene quantum dots. ACS Nano..

[24] Lin L, Zhang S (2012). Creating high yield water soluble luminescent graphene quantum dots via exfoliating and disintegrating carbon nanotubes and graphite flakes. Chem. Commun..

[25] Dong Y, Shao J, Chen C (2012). Blue luminescent graphene quantum dots and graphene oxide prepared by tuning the carbonization degree of citric acid. Carbon.

[26] Eda G, Lin Y-Y, Mattevi C (2010). Blue photoluminescence from chemically derived graphene oxide. Adv. Mater..

[27] Liu J, Luo XLH (2014). One-step preparation of nitrogen-doped and surface-passivated carbon quantum dots with high quantum yield and excellent optical properties. RSC Adv..

[28] Shen Y, Zhu X, Yang J, Zong JZ (2012). One-pot hydrothermal synthesis of graphene quantum dots surface-passivated by polyethylene glycol and their photoelectric conversion under near-infrared light. New J. Chem..

[29] Kwon W, Rhee SW (2012). Facile synthesis of graphitic carbon quantum dots with size tunability and uniformity using reverse micelles. Chem. Commun.

[30] Dimos K (2016). Carbon quantum dots: surface passivation and functionalization. Current Organic Chemistry.

[31] Huili F, Ji Z, Chen X, Cheng A (2017). A versatile ratiometric nanosensing approach for sensitive and accurate detection of Hg^2+^ and biological thiols based on new fluorescent carbon quantum dots. Analytical and bioanalytical chemistry.

[32] Yuan F, Yuan T, Sui L (2018). Engineering triangular carbon quantum dots with unprecedented narrow bandwidth emission for multicolored LEDs. Nature Communications..

[33] Zhang J, Wang H, Xiao Y, Tang J, Liang C, Li F, Dong H, Xu W (2017). A simple approach for synthesizing of fluorescent carbon quantum dots from tofu wastewater. Nanoscale Research Letters.

[34] He M, Zhang J, Wang H, Kong Y, Xiao Y, Xu W (2018). Material and optical properties of fluorescent carbon quantum dots fabricated from lemon juice via hydrothermal reaction. Nanoscale Research Letters.

[35] Peng J, Gao W, Gupta BK (2012). Graphene quantum dots derived from carbon fibers. Nano Lett.

[36] Zhu S, Zhang J, Tang S (2012). Surface chemistry routes to modulate the photoluminescence of graphene quantum dots: from fluorescence mechanism to up-conversion bioimaging applications. Adv. Funct. Mater.

[37] Niino S, Takeshita S, Iso Y, Isobe T (2016). Influence of chemical states of doped nitrogen on photoluminescence intensity of hydrothermally synthesized carbon dots. Journal of Luminescence.

[38] Singh VK, Singh V, Yadav PK (2018). Bright-blue-emission nitrogen and phosphorusdoped carbon quantum dots as a promising nanoprobe for detection of Cr(VI) and ascorbic acid in pure aqueous solution and in living cells. New J.Chem..

[39] Song Z, Quan F, Xu Y, Liu M, Liang C, Liu J (2016). Multifunctional N, S co-doped carbon quantum dots with pH- and thermo-dependent switchable fluorescent properties and highly selective detection of glutathione. Carbon..

[40] Zhu S, Song Y, Zhao X, Shao J, Zhang J, Yang B (2015). The photoluminescence mechanism in carbon dots (graphene quantum dots, carbon nanodots, and polymer dots): current state and future perspective. Nano Research.

[41] Yang YH, Cui JH, Zheng MT, Hu CF, Tan SZ, Xiao Y (2012). One step synthesis of amino-functionalized fluorescent carbon nanoparticles by hydrothermal carbonization of chitosan. Chem Commun..

[42] Li Y, Wang J, Li X (2012). Discharge product morphology and increased charge performance of lithium-oxygen batteries with graphene nanosheet electrodes: the effect of sulphur doping. Mater. Chem.

[43] Han Y, Tang D, Yang Y (2015). Non-metal single/dual doped carbon quantum dots: a general flame synthetic method and electro-catalytic properties. Nanoscale..

[44] Zhang F, Feng X, Zhang Y (2013). Photoluminescent carbon quantum dots as directly film-forming phosphor towards white LEDs. Nanoscale.

[45] Li C, Ding L, Liang C, Zhang J, Zhang C, Mei H (2017). Photon-induced light emission from foamed gold with micro/ nanohollow sphere structures. ACS Omega.

[46] Xu Q, Zhou Q, Zheng H, Xue Q (2013). Single-particle spectroscopic measurements of fluorescent graphene quantum dots. ACS Nano.

[47] Mei Q, Zhang Z (2012). Photoluminescent graphene oxide ink to print sensors onto microporous membranes for versatile visualization bioassays. Angew. Chem. Int. Ed.

